# Enhanced Insight into the Autoimmune Component of Glaucoma: IgG Autoantibody Accumulation and Pro-Inflammatory Conditions in Human Glaucomatous Retina

**DOI:** 10.1371/journal.pone.0057557

**Published:** 2013-02-25

**Authors:** Oliver W. Gramlich, Sabine Beck, Nadine von Thun und Hohenstein-Blaul, Nils Boehm, Anika Ziegler, Jan M. Vetter, Norbert Pfeiffer, Franz H. Grus

**Affiliations:** 1 Experimental Ophthalmology, Department of Ophthalmology, University Medical Center Mainz, Germany; 2 Cornea Bank of Rhineland-Palatine, Department of Ophthalmology, University Medical Center Mainz, Germany; Centre for Eye Research Australia, Australia

## Abstract

**Background:**

There is accumulating evidence that autoimmune components, such as autoantibodies and autoantibody depositions, play a role in the pathogenesis of neurodegenerative diseases like Alzheimeŕs disease or Multiple Sclerosis. Due to alterations of autoantibody patterns in sera and aqueous humor, an autoimmune component is also assumed in the pathogenesis of glaucoma, a common reason for irreversible blindness worldwide. So far there has been no convincing evidence that autoantibodies are accumulated in the retina of glaucoma patients and that the local immune homeostasis might be affected.

**Methods and Results:**

Six human glaucomatous donor eyes and nine samples from donors with no recorded ocular disease were included. Antibody microarrays were used to examine the patterns of pro-inflammatory proteins and complement proteins. Analysis of TNF-α and interleukin levels revealed a slight up-regulation exclusively in the glaucomatous group, while complement protein levels were not altered. IgG autoantibody accumulations and/or cellular components were determined by immunohistology (n = 4 per group). A significantly reduced number of retinal ganglion cells was found in the glaucomatous group (healthy: 104±7 nuclei/mm, glaucoma: 67±9 nuclei/mm; p = 0.0007). Cell loss was accompanied by strong retinal IgG autoantibody accumulations, which were at least twice as high as in healthy subjects (healthy: 5.0±0.5 IgG deposits/100 cells, glaucoma: 9.4±1.9 IgG deposits/100 cells; p = 0.004). CD27^+^ cells and CD27^+^/IgG^+^ plasma cells were observed in all glaucomatous subjects, but not in controls.

**Conclusion:**

This work provides serious evidence for the occurrence of IgG antibody deposition and plasma cells in human glaucomatous retina. Moreover, the results suggest that these IgG deposits occurred in a pro-inflammatory environment which seems to be maintained locally by immune-competent cells like microglia. Thereby, glaucoma features an immunological involvement comparable to other neurodegenerative diseases, but also shows a multifactorial pathomechanism, which diverges and might be linked to the specific nature of both eye and retina.

## Introduction

“*Losing your nerves? Maybe it’s the antibodies*.” This citation indicates the growing acceptance of neuronal reactive antibody (Ab) involvement in the pathogenesis of neurodegenerative diseases [1]. A first example is Myasthenia gravis (MG), in which autoantibodies against nicotinic acetylcholine receptor and muscle-specific tyrosin kinase inhibit the signal transduction at the neuromuscular junction, and additionally lead to an immune-mediated reduction of the receptor [2]. The resulting muscular atrophy, described in late stages of MG, is also known from Multiple sclerosis (MS) and associated with the degeneration of axons. MS is described as an autoimmune, primarily T-cell- mediated, inflammatory demyelination of the central nervous system (CNS), including the optic nerve [3], [4], [5]. Interestingly, recent studies discussed the involvement of antibodies in the pathomechanism of MS [6], characterized by the occurrence of autoreactive antibodies against components of the myelin sheath, like myelin-basic protein (MBP) [7], myelin-oligodendroglycoprotein (MOG) [8], or proteolipid protein (PLP) [9]. Similarly, Alzheimeŕs disease (AD), the leading cause for dementia [10], was suggested to have an autoimmune component [11]. The hallmarks of AD pathology are amyloid-β deposition in neurons, the so called amyloid plaques, and neurofibrillary tangles, resulting in progressive neurodegeneration [12], [13]. Up to now, several autoantibodies have been described in AD, featuring Abs against β-amyloid, S100, glial fibrillary acidic protein (GFAP), aldolase, microglia, several neurotransmitters, etc. [14]. These facts suggest a link between specific IgG autoantibody reactivity and neurodegeneration [15], [16], [17], [18], [19].

During the past 15 years, several studies on IgG antibody patterns in blood and aqueous humor revealed strong alterations in glaucoma patients as well [20], [21], [22], [23], [24], [25], [26], [27], [28], [29] and furthermore these disease-specific changes remained stable in different study populations [30]. Glaucoma, one of the most common causes of irreversible blindness worldwide [31], [32], is a neurodegenerative disease characterized by a progressive loss of retinal ganglion cells (RGCs) and their axons, which leads to a typical pattern of visual field loss in more advanced stages [33]. While the underlying pathogenesis is influenced by a heterogeneous group of ocular disorders, a high intraocular pressure is known as the major risk factor [34], [35]. In detail, mainly elevated autoantibody levels, but also decreased titers were found against a) several heat shock protein (HSP27, HSP60, HSP70) [36], [37], [38], b) some crystallines (α-A-, α-B) [36], [38], c) structural proteins like GFAP, vimentin [38], MBP [24], d) enzymes as γ-enolase [39] and neuron specific enolase [40] or glutathione-S-transferase [41], and e) others like anti-phosphatidylserine [42], glycosaminoglycans [43], α-fodrin [30], retinaldehyde-binding protein [44] and retinal S-antigen [44] in the sera and aqueous humour of glaucoma patients.

The changes of naturally occurring IgG autoantibody repertoires strongly implicate a role for autoimmunity in the neurodegenerative processes of glaucoma. Some of the autoantibodies found in glaucoma occurred in other neurodegenerative diseases as well, for example MPB in MS or GFAP in AD. Since Glaucoma is sometimes referred to as “ocular Alzheimer’s” [45], we wanted to address the question: Are there similar pathogenic circumstances in glaucoma as in AD or MS? The aspects of neuronal cell loss and alteration in the humoral immunity were met, but are IgG autoantibodies also accumulated in the damaged tissue, i.e. in glaucomatous retina? Furthermore, little is known about the local immunological conditions in the retina. In particular, it is unclear whether a local pro-inflammatory environment could facilitate an autoimmunological process, since the eye is considered an immune privileged site with a selectively anti-inflammatory and immunosuppressive homeostasis, in order to prevent potentially vision-impairing inflammation [46], [47]. Evidence for complement activation in glaucomatous retina via classical and lectin pathway has been gathered [48], but only limited hints for autoantibody occurrence in glaucoma patients were given by Wax et al. [49].

In the present study, we use an antibody microarray approach to examine the patterns of pro-inflammatory proteins such as INF-γ, TNF-α, several interleukins and protein levels of the complement cascade in retinal samples obtained from human donor eyes with or without glaucoma. Furthermore, this study aimed to prove the occurrence of IgG autoantibody accumulation in glaucomatous retina. Additionally, cellular components which are linked to i) secretion of IgG, ii) interaction with immunglobulins and iii) immune modulation like B-cells, T-cells and microglia were determined immunohistochemically using suitable markers in retinal cross-sections.

## Materials and Methods

### Donor Eyes

Donor eyes were obtained from the Cornea Bank of Rhineland-Palatine, Department of Ophthalmology, University Medical Center Mainz. The guidelines for corneal donations are regulated in the German Transplantation Law and are in agreement with the requirements of the Declaration of Helsinki, which were followed accordingly. Eyes were only used for this study, when there was explicit approval from the deceased’s relatives for experimental usage of remaining ocular donor tissue that was not needed for transplantation. Exclusion criteria for usage as donors were any kind of infectious ocular diseases, ocular tumors, neurological diseases (Multiple sclerosis, Alzheimeŕs disease, Parkinson, amyotrophic lateral sclerosis and Creutzfeldt-Jakob). Autoimmune diseases were also excluded as well as systemic viral, fungal and bacterial infections, hepatitis, HIV, malignant neoplasia and persons who required dialysis. History and ophthalmological background were requested from the closest relatives, general practitioner and the last attending doctor. The samples were grouped in ocular diseases like glaucoma, uveitis, etc., but specific classifications in glaucoma forms, such as primary open angle glaucoma or normal tension glaucoma could not be made. For age- as well as gender-matched groups, six samples from glaucomatous donors and nine samples from healthy subjects were selected for the study. Five adequate pairs were built individually for the antibody microarray analysis and due to the bad initial tissue conditions, four pairs were selected for the immunohistology. Out of all samples, one healthy and three glaucomatous samples were used in both approaches.

### Sample Preparation

All samples arrived semi-sterile without the cornea, unfixed and frozen in liquid nitrogen. First, the frozen eyes were sliced saggitally through the optic nerve head with a sterile hand saw and thawed. The retinas were dissected from one half of the bulbus, the blood vessels were removed and retinal tissues were prepared for the microarray approach as described below. After remodeling the eyes architecture of the other half of the bulbus, the tissues were fixed in 4% formaldehyde (Carl Roth, Karlsruhe, Germany) for 10 days. Then, samples underwent paraffin embedding automatically through Sakurás Tissue Tek auto TEC in a standard procedure containing fixation, dehydration, clearing and paraffination. 5 and 10 µm thick serial cross-sections were sliced on a rotation microtome (Biocut, Reichert &Jung), transferred onto Superfrost plus slides (Thermo scientific, Schwerte, Germany), dried and stored at 37°C until staining.

### Sample Treatment for Microarray Approach

Retinal tissues were homogenized with a pestle in a mortar under liquid nitrogen and resuspended in 100 µl sample buffer (0.1% SDS in Dulbecco’s PBS). After 20 minutes of sonification at 4°C, cell debris was discarded by centrifugation for 20 minutes at 2000 rpm at 4°C. Soluble protein extracts were subsequently centrifuged several times for 2 h at 14000 rpm at 4°C and the resulting pellets were discarded. A measurement of the total protein concentration was performed using a bicinchoninic acid protein assay (BCA kit, Thermo). To visualize protein levels in the retinal tissue samples, 2.5 µg and 5 µg of protein were labeled with 0.3 µl respectively 0.6 µl of an amine-reactive fluorescent dye (DyLight®649, Thermo). After incubation with the dye for 1 hour in the dark at room temperature, excessive fluorescent dye was inactivated by the addition of 100 µl glycine-solution (100 mM) to the sample solution. Labeled protein extracts were incubated subsequently on antibody microarrays (Preparation is described below).

### Antibody Microarray Preparation

Antibody microarrays were prepared by spotting commercially available purified antibodies on nitrocellulose coated slides (Oncyte, nitrocellulose 16 multi-pad slides, Grace Bio-Labs, Bend, USA) using a non-contact array spotter (sciFLEXARRAYER 3, Scienion, Berlin, Germany). For the analysis of cytokines and complement protein levels, Abs against IL-1β, IL-6, IL-8, (Biolegend, San Diego, CA, USA), TNF-α (BD BioSciences PharMingen, San Diego, CA, USA), IFN-γ (BD BioSciences PharMingen, San Diego, CA, USA), C1, C5, C6, C7, C8 (GeneTex, San Antonio, TX; USA) and C3 (HyCult Biotechnology BV, Uden, The Netherlands) were part of antibody setup, as well as anti-calcium binding adaptor molecule 1 as a microglia/macrophage marker (Iba 1, Wako Pure, Osaka, Japan) [50]. Two drops (each 250 pl) of each antibody were spotted in triplicates. For incubation with retina samples, slides were covered with 16-pad frame hybridization chambers (FAST; Whatman; Maidstone, UK). All incubation steps were performed at 4°C under slight agitation. First, the unspecific binding sites of the nitrocellulose membrane were blocked by incubation with 100 µl of non-fat-dry-milk (4% in PBS) for one hour. After washing the slides three times 10 minutes each time with 100 µl PBS-T (0.5% Tween 20 in PBS), labeled protein samples were incubated over night. Slides were washed again, 15 minutes with PBS-T followed by a two times washing step with ultra pure water and dried afterwards (Speed Vac; Thermo). Emitted fluorescence signals were digitized by scanning the slides with a high-resolution confocal scanner and spot intensities were quantified (Imagene 5.5, BioDiscovery Inc., Los Angeles, California, USA). For further analysis the local background signal was subtracted from the median signal of each spot followed by averaging the three technical replicates for each antibody. For data analysis, the raw data were globally normalized by using a constant scale factor for each subarray. The resulting normalization coefficients were applied to each intensity value yielding a relative intensity (U) for each antibody reactivity. Performing a conservative statistical analysis, the triplicate values were averaged and the mean values of each subject underwent student’s t-test for a group-specific comparison. Due to the sample size and the multiple t-test scheme, data were interpreted for trends in consideration of the p-values and are presented as mean values of the relative intensity (U) ± standard deviation (mean ± SD). Additionally, the cumulative levels as ∑ of the relative intensity of all used Abs for complement (and the pro-inflammatory components) were calculated as a basic “first glance” parameter, since activation of the complement system in ocular tissues seems to require general accumulation of all important complement proteins [48].

### IgG Autoantibody Staining and Cell Quantification

Procedure for IgG autoantibody staining was performed as previously described [51], [52]. In detail, slices were dewaxed in xylene three times and rehydrated in a sequential ethanol gradient (100%, 100%, 96%, 96%, 70%, each 5 min) to Aqua dest. Antigen retrieval was performed in preheated Target Retrieval Solution (DAKO, Hamburg, Germany) at 78°C for 45 min, followed by short washes in PBS (Dulbecco’s PBS, Sigma Aldrich, St. Louis, MO, USA). Endogenous peroxidase was quenched through incubation in 0.3% H_2_O_2_/PBS for 10 min. The sections were then blocked with 1% normal goat serum/1% bovine serum albumin/PBS for 10 min, afterwards rinsed in PBS and subsequently incubated with a horseradish peroxidase (HRP) conjugated rabbit anti-human IgG (H+L, 1∶200, DAKO) for 3 h at room temperature (RT). 3.3-diaminobenzidine-tetrahydrochloride (DAB, DCS, Hamburg, Germany) reaction was performed on slide for 7 min after three washes in PBS, followed by hematoxilin (Merck, Darmstadt, Germany) counterstaining. Negative controls were conducted through incubation of equally treated slides with DAB, without anti-IgG HRP antibody incubation.

The number of remaining cells in the retinal ganglion cell-layer (rgcl) and IgG autoantibody deposition were evaluated in three serial sections (10 µm) of each sample (n = 4 per group). Of each section, 10 representative photographs (405×306 µm) were taken, using 20× magnifications of an Olympus Vanox T microscope (Olympus, Hamburg, Germany). The retinal length, the number of hematoxilin stained cells in the rgcl, as well as the number of IgG^+^ cells were counted later by two independent observers masked to the protocol. Repetitions, performed to ensure reproducibility, revealed reliable results. Endothelial cells were excluded from further analysis, while the number of cells in the rgcl was calculated per mm retina by the total retinal length of all ten frames. Simultaneously, numbers of distinct IgG autoantibody deposits were evaluated per 100 cells in the rgcl. An averaged data set of the two independent observers was used for final analysis, for which the mean values were built from the three slides of each sample and student’s t-test was applied. P-values <0.05 were considered as significant.

For the immunofluorescent approach to visualize IgG deposits, the slides were incubated with a FITC-labeled rabbit anti-human IgG antibody (1∶200, GeneTex) or goat anti-human IgG FITC (1∶40 abcam, Cambridge, UK) for 3 h at RT, washed with PBS and mounted with diamidin-2-phenylindol (DAPI) supplemented Vectashield (Vector, Burlingame, CA, USA). The IgG autoantibodies were visualized with a Nikon TE 2000 microscope (Nikon, Düsseldorf, Germany) equipped with a highly sensitive CCD camera and Nikońs Lucia G/F software. A high resolution fluorescence microscope, Keyence BZ-9000 (Neu-Isenburg, Germany), was also employed to acquire a higher depth of sharpness via multi-focus of z-stack images and digital image editing.

### T-cell, B-cell and Microglia Immunostaining

Several antibodies (Abs) were chosen to identify the cellular immune components in the retinas. A minimum of five cross-sections (5 µm) from each sample (n = 4 per group) were immunohistochemically and immunofluorescently stained individually for each primary antibody as described above. Furthermore, additional sections were processed for double immunofluorescence of IgG autoantibody depositions and CD markers mentioned below. Co-localization was examined using the “co-localization highlighter” plug in ImageJ software ((NIH, http://rsb.info.nih.gov/ij/). To investigate the possibility of an interaction between IgG and microglia, Iba 1/IgG immunostaining was implemented as described previously [53].

In detail, the following primary Abs were used in this study: rabbit anti-CD 3 (1∶200, 3 h RT, Linaris, Dossenheim, Germany), FITC conjugated anti-CD 3 (1∶200, 3 h RT, abcam), mouse anti-CD 20 (1∶200, 3 h RT, abcam), rabbit anti-CD 27 (1∶200, 3 h RT, abcam), FITC conjugated anti-CD 27 (1∶200, 3 h RT, BD Pharmingen), subsequently followed by specific secondary Abs: goat anti-rabbit IgG (H+L) Cy3 (1∶200, 90 min RT, Linaris), goat anti-rabbit IgG (H+L) HRP (1∶200, 90 min RT, Calbiochem, Merck), goat anti-mouse IgG F(ab) HRP (1∶200, 90 min RT, Abnova, Heidelberg, Germany) and goat anti-mouse IgG FITC (1∶200, 90 min RT, Abnova). Cross reactivity with rodents was known for the primary antibodies, so rodent brain, spleen and lymph nodes served as positive controls in every staining cycle. Negative controls were performed by single incubation of the secondary antibodies without previous primary antibody incubation.

## Results

### Increased Levels of Pro-inflammatory Cytokines in the Human Glaucomatous Retina

We were able to detect complex patterns of protein levels in retinas of glaucomatous and healthy subjects (n = 5 per group) using antibody microarray technique. Both used total protein amounts (2.5 µg or 5 µg) provided similar protein profiles after normalization. For measurement of protein level in the low intensity range, such as IL-8 or C3, the microarray signal detection was more sensitive using a total protein concentration of 5 µg and thus considered for further analysis. Regarding the analyzed proteins of the complement cascade, we found a heterogenic and individual distribution of distinct complement proteins in both study groups. In detail, retinal amounts of C1, C2 and C8 were occasionally increased in the control samples (C1: ctrl = 64139±8236 U, glaucoma = 49508±21343 U, p = 0.19; C2: ctrl = 46938±11073 U, glaucoma = 40530±8140 U, p = 0.33; C8: ctrl = 52926±5258 U, glaucoma = 41533±8877 U, p = 0.03). And, vice versa, C3 and C6 were increased in the glaucomatous group (C3: ctrl = 6122±527 U, glaucoma = 8884±2106 U, p = 0.02; C6: ctrl = 32254±10387 U, glaucoma = 51599±9439 U, p = 0.01), while the level of C5 was equal in both groups (C5: ctrl = 43163±18772 U, glaucoma = 44443±155244 U, p = 0.9). In summary, the analysis showed some highly different regulations of increased as well as decreased levels in glaucoma, but without any specific or pathogenetic pattern. Furthermore, the cumulative level (U of ∑ C1, C2, C3/3b, C5, C6, C8) of complement proteins showed no differences between the groups (∑ctrl: 245832 U, ∑glaucoma: 236488 U).

**Figure 1:**
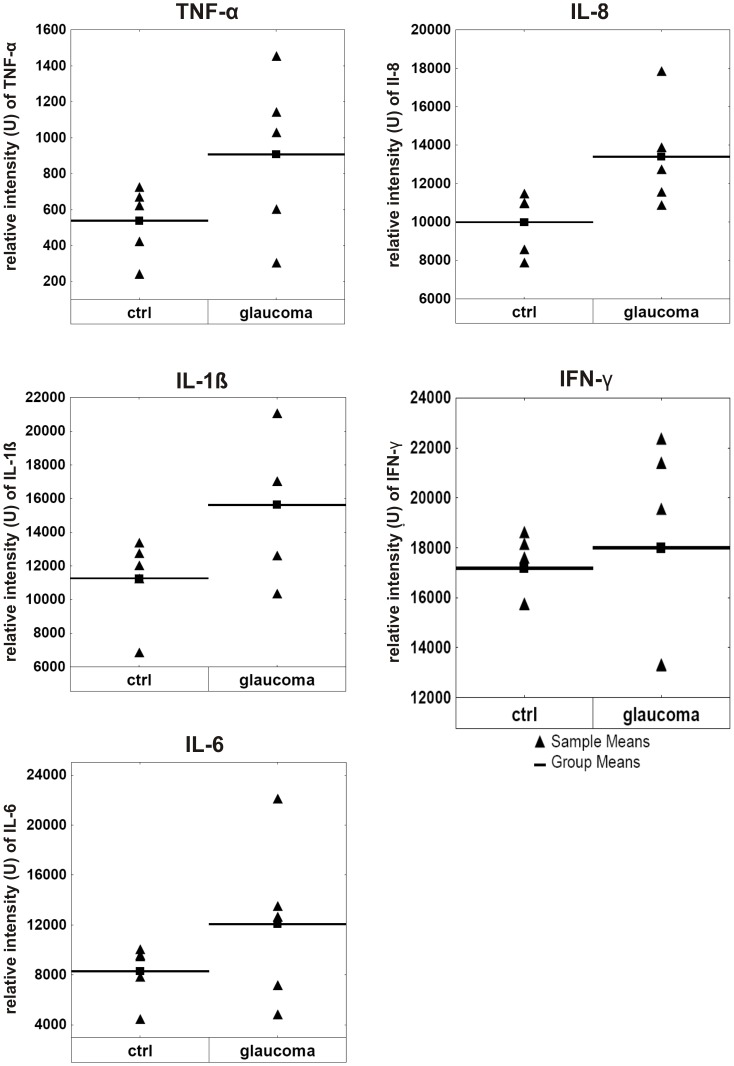
Levels of pro-inflammatory cytokines in glaucomatous retinal tissue. Figure 1 depicts the altered concentrations of the pro-inflammatory cytokines TNF-α, IL-1ß, IL-6, IL-8 and IFN-γ in detail. The arrowheads indicate the relative intensity (U) on the y-axis of each individual subject as sample means and the black bar summarizes the group means. All investigated pro-inflammatory cytokines were up-regulated in the glaucomatous group (glaucoma) compared to the healthy subjects (ctrl). (In some box-plots, the arrowheads of sample means overlaid.).

The analysis of TNF-α and interleukin levels revealed a more uniform and consistent result with a slight up-regulation exclusively in the glaucomatous group. An increased protein level was measureable for TNF-α (TNF-α: ctrl = 538±200 U, glaucoma = 907±454 U; p = 0.13) and the interleukins IL-1ß (IL-1β: ctrl = 11262±2590 U, glaucoma = 15625±4194 U; p = 0.08) and IL-6 (IL-6: ctrl = 8301±2287 U, glaucoma = 12073±6695 U; p = 0.26). The retinal protein amount measured for interleukin 8 noted a large increase in glaucoma subjects (IL-8: ctrl = 9990±1632 U, glaucoma = 13394±2749 U; p = 0.04). Regarding the level of IFN-γ no group differences could be detected (IFN-γ: ctrl = 17189±1346 U, glaucoma = 18003±4390 U; p = 0.7). To get an enhanced insight into regulation of the relevant proteins, the relative intensities (U) of all samples per group are shown for TNF-α, IFN-γ, IL-1β, IL-6 and IL-8 in figure 1. The cumulative level of pro-inflammatory proteins (U of ∑ TNF-α, IFN-γ, IL-1β, IL-6, IL-8) is enhanced of approx. 25% in glaucomatous retina (∑ctrl: 47281 U, ∑glaucoma: 60004 U).

The analysis of Iba 1 showed increased levels of this microglia/macrophage marker in the glaucoma group (Iba1: ctrl = 9725±3941 U, glaucoma = 12602±6406 U).

### IgG Autoantibody Accumulation, Cell Loss and Hints for Cellular Components in Glaucomatous Retina

**Figure 2:**
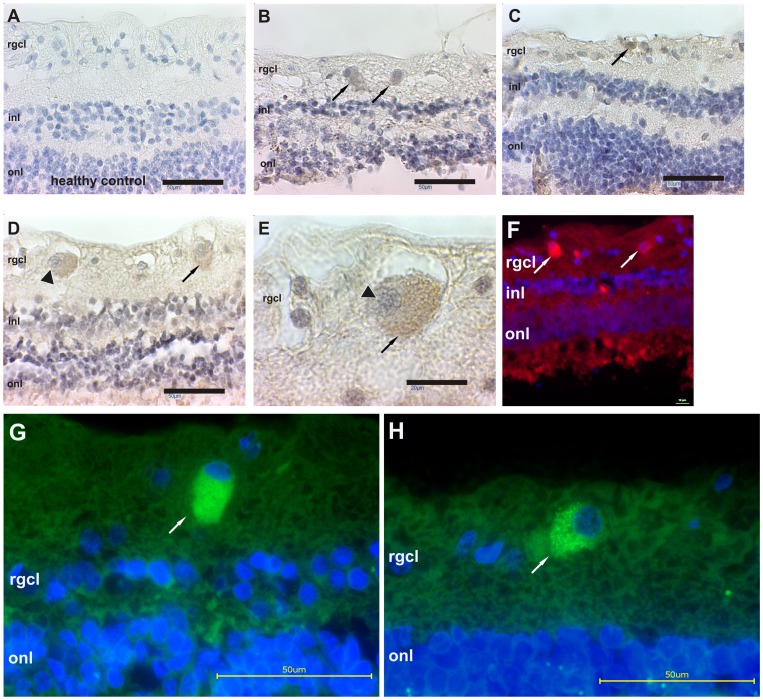
IgG autoantibody accumulates in glaucomatous retina. The image in **A** representatively shows the retinal morphology of healthy control subjects, characterized by an intact retinal architecture, comparatively large numbers of cells in the retinal ganglion cell-layer (rgcl) and few IgG depositions. In the glaucomatous subjects, we examined IgG positive immunoreactions by brown DAB staining (indicated by arrows) and were able to distinguish three major types of IgG accumulations. In **B**, IgG depositions are fuzzy, scattered and seem to be located somatically, intra- and extracellularly in presumably neurons (type 1). As depicted in **C**, more intensely stained, compact and smaller IgG depositions (type 2) were located on fragmented nuclei of apparently apoptotic neurons. The third type of IgG deposition in **D** (arrowhead) was larger than the other depositions (arrow), usually round or oval and the nucleus size was increased nearly twofold compared to the majority of the remaining nuclei in the rgcl. These cells showed characteristics of plasma cells with eccentric and heterochromatic nuclei, enhanced size, cartwheel or clock face morphology and immunoglobulin presence, as indicated in **E** (100× magnification of the arrowhead marked cell in **D**). This cell type was investigated further with appropriate CD markers to elucidate their origin. The image shown in **F** represents our immunofluorescent visualization of IgG antibody accumulations (arrows, red fluorescence) and detailed multi-focal images were acquired subsequently confirmed by different anti-human IgG antibodies in the following images **G** and **H**. A homogenous, cloudy IgG accumulation (green fluorescence) is shown in **G**, whereas scattered punctuated IgG dots could be seen in **H**. Scale bars in **A–D, G and H** are 50 µm, in **E** 20 µm and in **F** 10 µm. In **F, G and H**, DAPI was used as nuclei dye (blue). Abbreviations: inl: inner nuclear layer, onl: outer nuclear layer.

We could successfully demonstrate the presence of IgG autoantibody accumulation in glaucomatous retina. But notably, we also detected some IgG autoantibody accumulations in the control subjects. Interestingly, three major types of IgG depositions were morphologically distinguishable and IgG accumulations could exclusively be found in the retinal ganglion cell layer, while few or only faint accumulations and diffuse staining were observed in other layers. As shown in figure 2, one pattern for IgG depositions (type 1) appeared cloudy scattered with punctuated dots, located in the soma of presumably retinal ganglion cells (figure 2 B, G, H). These punctuated dots of IgG accumulation were clearly identified at higher magnification after multi-focal image acquisition, but whether they are located intra- or extracellularly could not be evaluated (figure 2 H). Other IgG accumulations (type 2) were small, compact and more intensely stained on presumably apoptotic RGC with clearly fragmented nuclei (figure 2 C). The third type tended to be larger than the first, usually round or oval and was accompanied by a different type of nuclei, which was almost twice as large as the majority of cell nuclei in the rgcl (figure 2 D, E). These nuclei were mainly eccentric with heterochromatin and showed characteristics of plasma cells: the cartwheel or the clock face. Because this type of cell nuclei was only present in glaucomatous samples and did not always co-localize with IgG autoantibodies, we performed subsequent immunostaining with several CD markers. Additional positive controls for IgG were not necessary, because remaining blood in retinal vessels can easily be distinguished by morphology, especially of the endothelial cells, and always showed a positive IgG staining in both groups.

**Figure 3:**
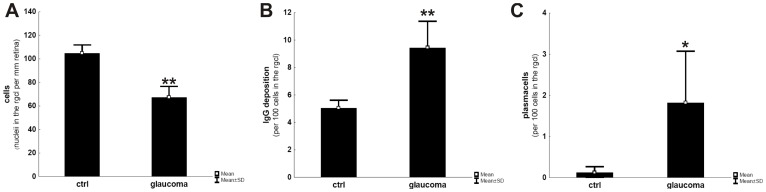
Quantification of cell loss, IgG depositions and plasma cells in cross-sections of glaucomatous and healthy retina. After immunostaining for IgG accumulations and hematoxylin counterstaining, as shown in figure 2, calculation of remaining neuronal cells in the retinal ganglion cell-layer to the retinal length revealed a significant lowering in the glaucomatous group compared to controls (**A**), while the number of IgG deposits in relation to the number of remaining cells is significantly increased (**B**). Based on morphological features, plasma cells were only detectable in the glaucomatous group as indicated in **C**. * = p<0.05; ** = p<0.01.

For analysis, a blinded observer was trained to distinguish the three types of IgG accumulation and sub-divide them into IgG depositions on neurons (type 1 and 2) and IgG accumulation in plasma cells (type 3). When analyzing the amount of cells after hematoxylin counterstaining, the glaucomatous group showed approx. 35% less nuclei in the rgcl. In n = 4 retinae per group, 104±7 (mean ± SD) nuclei were counted in the rgcl per mm for the controls compared to 67±9 nuclei in the glaucomatous samples, indicating a significant cell loss (p = 0.0007, figure 3 A). In the retina of glaucoma patients, the number of distinguishable IgG deposits ranged at 9.4±1.9 IgG deposits per 100 cells in the rgcl and thus was approximately twice as high as in healthy subjects (5.0±0.5 IgG deposits per 100 cells); statistics revealed a significant increase with p = 0.004 (figure 3 B). An occurrence of plasma cells based on IgG^+^ staining and characteristic morphological features was exclusively found in glaucoma tissues with a frequency of 1.8±1.2 per 100 cells in the rgcl (ctrl: 0.12±0.14 plasma cells per 100 cells) and therefore, revealed also a significant p-value with p = 0.03 (figure 3 C).

### Identification of Cellular Components: Immunohistological Evidence for T-cells, B-cells, Plasma Cells and Microglial Interactions

**Figure 4:**
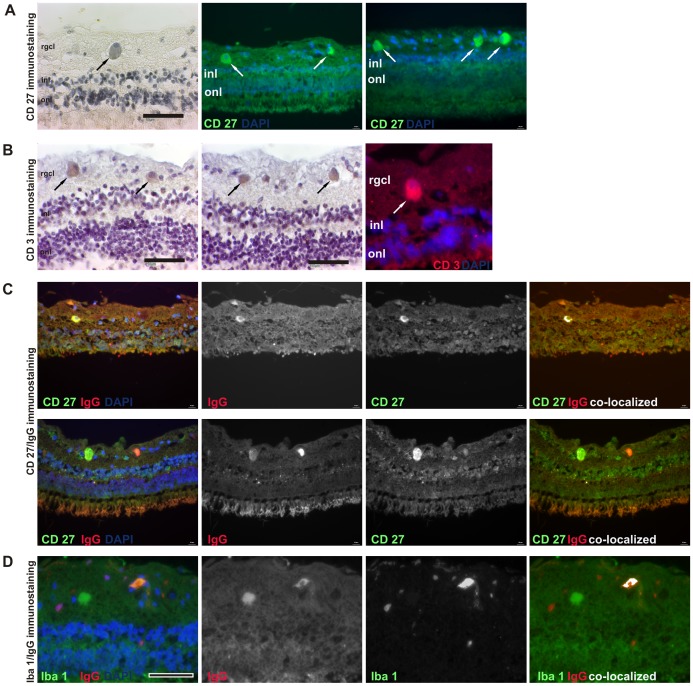
Evidence for B- and T-cells in glaucomatous retina. After immunodetection with markers against CD27 (panel **A)** and CD3 (panel **B)**, we detected several CD27^+^ cells in the rgcl of 4/4 glaucomatous retina as well as few CD3^+^ cells in one glaucoma subject by brown DAB deposition (indicated by the black arrows, scale bars 50 µm). Positive detection (white arrows, scale bars 10 µm) could be provided by the immunofluorescence approach for both CD markers in the rightmost images, while additional CD20 immunostaining in retina was always negative. Panel **C** shows the analysis of co-localization of CD27^+^ and IgG. As indicated in the rightmost images, the co-localized pixels of the individual fluorescence images for CD27 and IgG (middle images) were highlighted in white and identify this cell type clearly as a plasma cell. Furthermore, panel **D** depicts the co-localization of an IgG deposition in close contact to iba1^+^ microglia. The interaction between microglia and IgG could be understood functionally in relation to an opsonization. DAPI (blue): nuclei. Scale bars in panel C is 10 µm and in D 50 µm.

For analysis of cellular components we used markers against CD3, CD20 and CD27 in single or double-immunofluorescence labeling techniques (see table 1). All markers were tested for their specificity previously and rodent tissues (lymph nodes and spleen) served as positive controls throughout all stainings. A positive immunostaining for CD 20 was not detected in any used human retina and indicates the absence of naïve B-cells. Importantly, in 5/5 staining cycles, we observed CD27^+^ cells in all glaucomatous subjects, but not in controls (n = 4 per group, figure 4). As known from literature, CD27 is expressed on T-cells, memory B-cells and also on plasma cells [54], [55], [56], [57]. Because of the observation of CD3^+^ T-cells in some glaucomatous retinas (figure 4), we were not able to provide accurate classification based on CD27 immunostaining alone. To overcome this problem, we performed double- immunofluorescence stainings against CD27+CD3 (indicative of T-Cells) and CD27+IgG (indicative of plasma cells) [57], [58], [59], [60], [61]. For both immunostaining methods, we were able to detect CD27^+^/IgG^+^ and CD27^+^/CD3^+^ cells in the positive control tissues. In the retina of glaucoma patients, CD27/CD3 immunostaining was consistently negative and we only found CD27^+^/IgG^+^ cells (figure 4).

**Table 1:** Summary of immunostainings.

	IgG^+^	CD3^+^	CD20^+^	CD27^+^	CD27^+^/CD3^+^	CD27^+^/IgG^+^
**healthy (n = 4)**	+	−	−	−	−	−
**glaucoma (n = 4)**	++	+ (2/4)	−	++	−	+
**positive control** **(lymph nodes,** **spleen)**	++	++	++	++	+	+

Beside IgG accumulation, no detection of CD3, CD20 or CD27 positive cells were made in retina of healthy subjects. Glaucomatous retina revealed thereby evidences of CD27^+^ cells, CD27^+^/IgG^+^ plasma cells and few hints for T-cells in two of four glaucomatous samples.

Surprisingly, the number of CD27^+^/IgG^+^ plasma cells was not as high as expected and at lower frequency than the two CD27^+^ cells per 100 cells in the rgcl (see figure 3). Thus, there were more CD27^+^ cells in glaucomatous retina than CD27^+^/IgG^+^ plasma cells. This suggests a further mechanism beyond IgG production.

For the sake of completeness, the double-immunostaining was performed also on the (in previous single staining CD27-negative) tissues from healthy donors without any positive detection as illustrated in table 1.

After double-immunostaining of IgG and iba1, sporadic interactions between IgG and microglia cloud be examined exclusively in the retinal ganglion cell layer of glaucomatous subjects (figure 4). Typically, microglia covered small IgG deposits completely, indicating phagocytosis, or appeared in clusters around bigger IgG deposits.

## Discussion

The results of this study suggest features of AD´s pathology in glaucoma, like neuronal cell loss and alteration of the humoral immunity. Our data provide convincing evidence for the occurrence of IgG antibody deposition in a pro-inflammatory environment in glaucomatous retinas. The involvement of the complement system in the pathology of glaucoma, as described by Tezel et al. [48], who found a potential deficiency in intrinsic regulation of complement activation and a down-regulation of complement factor H (CFH), could not completely be confirmed in the presented study through our microarray approach, but also cannot be ruled out. The cumulative level of complement proteins in the retina did not differ between glaucoma and healthy subjects in our analysis and therefore, complement activation seemed to play a less important role. There is at most a hint to the alternative pathway in complement activation due to the increased level of C3 in glaucoma (p = 0.02). Looking at individual patient´s data (including values of both used protein concentrations, and data from the pilot study of this project), the complement proteins tended to be slightly increased in single glaucoma samples. But these are interpretations without any statistical validation and we assume a possible activation of the complement system in individual glaucoma cases, rather than in every glaucoma patient.

On the other hand, our analysis of pro-inflammatory components in the retina was relatively similar to findings of other studies investigating inflammatory cytokine expression in the aqueous humor of glaucomatous eyes. The concentrations of various interleukins have been found to be significantly up-regulated in these examinations. In particular, CXCL8 (IL-8) and VEGF were increased in aqueous humour and the pattern of IFN-γ, IL-12, and CXCL9 (MIG, Monokine induced by Gamma-Interferon) concentrations also suggest a local pro-inflammatory cellular response [62], [63]. We also observed comparable trends for pro-inflammatory regulations in glaucomatous retinal tissues in our study like elevated concentrations of TNF-α (p = 0.13), IL-1β (p = 0.08), IL-6 (p = 0.26) and IL-8 (p = 0.04) compared to non-glaucoma controls. The idea of a pro-inflammatory environment in the aqueous humor of glaucoma patients is not new, but importantly, we were able to confirm that there are similar circumstances in the retina, where the neurodegeneration takes place. The cumulative level of pro-inflammatory proteins is enhanced by approx. 25% in the glaucomatous retina. Despite the fact that p-values are below statistical significance, a clear trend was seen and the regulation of the different pro-inflammatory chemokines may well be relevant to the neurodegenerative orchestra. Independent of statistical significance, even small changes in the titer of only one component may alter the retinal homeostasis with strong impact. Further evidence for retinal alteration is given, as we demonstrate convincing data for the occurrence of IgG antibody accumulations, sporadic T-cells and plasma cells for the first time. Although Wax suggested their existence, he stated that their search in glaucomatous retina, as well as in Experimental Autoimmune Glaucoma animal model, might be unproductive [64]. Notably, we identified CD27^+^/IgG^+^ plasma cells. Interestingly, a subpopulation of the plasma cells was only CD27^+^ and negative for IgG. We suggest that this subpopulation contains IgM Abs instead of IgG or might be memory B-cells. The fact that we could not detect a co-localization of CD27 staining with T-cell specific CD3 marker leads us to the conclusion that the CD27^+^ cells are unlikely to be T-cells. Due to the absence of T-cells, the question arises which cell type maintains the pro- inflammatory conditions in glaucomatous retina and aqueous humor. CD27^+^ cells could be ruled out, because only IgM and IgG Abs but not cytokine secretion is restricted to the CD27^+^ B- lymphocyte subset [65]. We hypothesize that microglia might be responsible for cytokine secretion, as their potential of chemokine release in retinal degeneration has been described [66] and strong evidence was also given in our experimental autoimmune glaucoma animal model. In this model, we elicited a glaucomatous-like damage with a loss of retinal ganglion cells in Lewis rats by systemic immunization of a complex mixture of proteins obtained from the optic nerve or from the retinal ganglion cell-layer. The immunization led to an antigen specific, complex systemic immune response, which included the development of autoreactive antibodies against retinal and optic nerve epitopes with an increasing and time-dependent severity. The loss of RGCs in this model is accompanied by IgG autoantibody accumulation on cells in the ganglion cell layer and often found in co-localization with activated microglia cells [52], [53].

Several investigations support our hypothesis of microglial involvement, showing that HLA-DR^+^ microglia in the human glaucomatous optic nerve head occurred more frequently and these microglia contained increased amounts of TNF-α and its TNF-R1 receptors, as well as abundant levels of the growth factor TGF-ß [67], [68], [69]. In glaucomatous retinas, Tezel et al. observed a 20% up-regulation in number and size of HLA-DR^+^ microglia, mostly found in the inner part of the retina [70]. These findings were analogous to our results, as the microarray approach revealed increased retinal protein levels of almost one quarter of the microglial marker iba1 in our glaucoma patients (iba1: ctrl = 9725±3941 U, glaucoma = 12602±6406 U). Furthermore, interaction of microglia and IgG was examined by immunostaining and the numbers of Iba1^+^ microglia cells were slightly increased in the glaucoma group. In particular, amoeboid (activated) microglia occurred in clusters around larger IgG depositions in the retinal ganglion cell layer of glaucoma patients while healthy retina contained predominantly ramified (quiescent) and less microglia. As shown in figure 4, iba1^+^ microglia covers IgG, and trigger microglia activation by means of opsonization, regardless of whether this IgG deposit underwent phagocytosis or not. It is widely accepted that microglia get activated by interaction with their Fc receptors to opsonized antigens and respond with phagocytosis, antibody-dependent cell-mediated cytotoxicity (ADCC), and oxidative bursts [71], [72], [73]. Furthermore, microglial ADCC is a common feature of the pathogenesis of several neurodegenerative diseases [74], [75], [76]. We suggest that microglia activation might provide the increased levels of TNF-α and IL-8 in glaucomatous retina after activation by autoantibody depositions, maintain ADCC and regulate the pro-inflammatory homeostasis along with the recruitment of plasma cells in a kind of vicious circle.

Despite of some common features in the pathology of AD and glaucoma, we would not claim that glaucoma is ocular AD, because one major hallmark of AD is not met so far: the general occurrence of β-amyloid in glaucomatous retina. But we cannot exclude that β-amyloid might play a role in glaucoma or might be present in the retina. We further assume that in some cases of glaucoma retinal β-amyloid might be detectable and might influence disease progression, but does not represent a characteristic or essential feature in glaucoma pathogenesis. We understand this more like a concomitant phenomenon, indicating similarities in neurodegeneration as revealed by aqueous humour analysis [77]. Moreover, glaucoma features different aspects of other neurodegenerative diseases, such as serum antibodies against the myelin sheath like in MS [24] and identical serum biomarkers can be found in different neurodegenerative diseases [14], [78]. For example nitric oxide and mitochondrial dysfunction are major pathogenetic mechanism in various CNS diseases, and also in glaucoma [79], [80]. The alteration of autoantibody patterns in glaucoma is possibly a consequence of the immune response after cleaning and healing [81] of retinal events, but it is unclear if and which autoantibody reactions may be causative in an autoimmune context and which are an epiphenomenon. Nevertheless, autoantibodies seem to be involved in the pathology of glaucoma, but if these specific depositions found in the retina contribute to the pathological features of classical neurodegenerative diseases has to be further investigated. Additionally, some pathological components of glaucoma were not found in other neurodegenerative diseases like fluctuation in the pressure system i.e. elevated intraocular pressure, specific structural alterations i.e. the laminar cribosa or ocular blood flow alterations, interruption of the neurotrophic transport in the optic nerve or neuronal damage by light with specific conditions for oxidative stress and glutamate toxicity in the retina [82], [83], [84], [85].

In conclusion, autoimmune components such as autoantibodies and autoantibody depositions play a role in the pathogenesis of various neurodegenerative diseases. In glaucoma, one of the most common reasons for blindness worldwide, strong alterations in autoantibody profiles were found in glaucoma patients [21], [23], [24], [25], [26], [27], [28], [29], [36], [37], [86], [87] and indicate also an autoimmune involvement. But the pathogenetic mechanisms of these altered autoantibodies are widely unknown. Therefore we examined glaucomatous tissues for congruities and differences to pathological mechanisms involving autoantibodies in other neurodegenerative diseases like AD and MS. In this study we could show, that in glaucoma, IgG autoantibodies are also accumulated in the neurodegenerating tissue: the retina. Moreover, the IgG autoantibody deposits are accompanied by CD27^+^/IgG^+^ plasma cells and occurred in pro-inflammatory conditions with increased levels of TNF-α, IL-6 and IL-8. These are comparable circumstances as in other neurodegenerative diseases and the secretion of such chemokines seems be maintained by microglia. Besides these congruent autoimmune features, the pathology of glaucoma also involves alternative and multifactorial pathomechanisms, which are linked to the specific nature of the eye and the retina.
